# Whole bone testing in small animals: systematic characterization of the mechanical properties of different rodent bones available for rat fracture models

**DOI:** 10.1186/s40001-018-0307-z

**Published:** 2018-02-14

**Authors:** Peter M. Prodinger, Peter Foehr, Dominik Bürklein, Oliver Bissinger, Hakan Pilge, Kilian Kreutzer, Rüdiger von Eisenhart-Rothe, Thomas Tischer

**Affiliations:** 1 Klinik für Orthopädie und Sportorthopädie, Klinikum rechts der Isar der Technischen Universität München, Ismaninger Straße 22, 81675 Munich, Germany; 2 Abteilung für Biomechanik, Klinik für Orthopädie und Sportorthopädie, Klinikum rechts der Isar der Technischen Universität München, Munich, Germany; 3 Abteilung für Fuß- und Sprunggelenkchirurgie, Klinik Volkach, Volkach, Germany; 4 Klinik für Mund-, Kiefer- und Gesichtschirurgie, Klinikum rechts der Isar der TU München, Munich, Germany; 5 Orthopädische Klinik, Universitätsklinikum Düsseldorf, Heinrich-Heine-Universität, Düsseldorf, Germany; 6 Klinik für Mund-, Kiefer- und Gesichtschirurgie, Universitätsklinikum Hamburg Eppendorf, Hamburg, Germany; 7 Orthopädische Klinik und Poliklinik der Universität Rostock, Rostock, Germany

## Abstract

**Objectives:**

Rat fracture models are extensively used to characterize normal and pathological bone healing. Despite, systematic research on inter- and intra-individual differences of common rat bones examined is surprisingly not available. Thus, we studied the biomechanical behaviour and radiological characteristics of the humerus, the tibia and the femur of the male Wistar rat—all of which are potentially available in the experimental situation—to identify useful or detrimental biomechanical properties of each bone and to facilitate sample size calculations.

**Methods:**

40 paired femura, tibiae and humeri of male Wistar rats (10–38 weeks, weight between 240 and 720 g) were analysed by DXA, pQCT scan and three-point-bending. Bearing and loading bars of the biomechanical setup were adapted percentually to the bone’s length. Subgroups of light (skeletal immature) rats under 400 g (*N* = 11, 22 specimens of each bone) and heavy (mature) rats over 400 g (*N* = 9, 18 specimens of each bone) were formed and evaluated separately.

**Results:**

Radiologically, neither significant differences between left and right bones, nor a specific side preference was evident. Mean side differences of the BMC were relatively small (1–3% measured by DXA and 2.5–5% by pQCT). Over all, bone mineral content (BMC) assessed by DXA and pQCT (TOT CNT, CORT CNT) showed high correlations between each other (BMC vs. TOT and CORT CNT: *R*^2^ = 0.94–0.99). The load–displacement diagram showed a typical, reproducible curve for each type of bone. Tibiae were the longest bones (mean 41.8 ± 4.12 mm) followed by femurs (mean 38.9 ± 4.12 mm) and humeri (mean 29.88 ± 3.33 mm). Failure loads and stiffness ranged from 175.4 ± 45.23 N / 315.6 ± 63.00 N/mm for the femurs, 124.6 ± 41.13 N / 260.5 ± 59.97 N/mm for the humeri to 117.1 ± 33.94 N / 143.8 ± 36.99 N/mm for the tibiae. Smallest interindividual differences were observed in failure loads of the femurs (CV% 8.6) and tibiae (CV% 10.7) of heavy animals, light animals showed good consistency in failure loads of the humeri (CV% 7.7). Most consistent results of both sides (left vs. right) in failure loads were provided by the femurs of light animals (mean difference 4.0 ± 2.8%); concerning stiffness, humeri of heavy animals were most consistent (mean difference of 6.2 ± 5%). In general, the failure loads showed strong correlations to the BMC (*R*^2^ = 0.85–0.88) whereas stiffness correlated only moderate, except for the humerus (BMC vs. stiffness: *R*^2^ = 0.79).

**Discussion:**

Altogether, the rat’s femur of mature specimens showed the most accurate and consistent radiological and biomechanical results. In synopsis with the common experimental use enabling comparison among different studies, this bone offers ideal biomechanical conditions for three point bending experiments. This can be explained by the combination of a superior aspect ratio and a round and long, straight morphology, which satisfies the beam criteria more than other bones tested.

**Electronic supplementary material:**

The online version of this article (10.1186/s40001-018-0307-z) contains supplementary material, which is available to authorized users.

## Background

Rodent models are still an important issue in the preclinical research of bones. They provide insights into bone metabolism of living organisms and potentially uncover positive or negative effects in the use of certain types of medication [1–4]. Furthermore, they allow us to investigate the physiological process of bone healing. Because of their surgical feasibility and standardisation, rat fracture models are of major significance [5].

Experimental bones can be analysed by morphological and functional trials. Radiological procedures such as pQCT (peripheral quantitative computed tomography), Micro-CT and DXA (dual energy X-ray absorptiometry) are useful measurements to evaluate the microstructure, the density and the mineral content of calcified tissue [1, 6–9]. Additionally geometric parameters can be calculated and compared. Cellular analyses and even dynamics in bone apposition can be visualized by histological staining [10]. Thus, only functional trials using destructive fracture tests provide the most accurate answers to the stability of the subjected bone [11].

Common biomechanical testing protocols included compression, torsional, tensile, four-point bending and three-point bending tests [5, 12, 13]. Because of its simple and reproducible setup, three-point bending is a frequent and prevalent method of mechanical testing in small animal fracture models [5, 8, 12, 14].

Reviewing the recent literature, we surprisingly concluded that systematic tests/research on inter- and intra-individual differences of common rat bones examined, are not available for the most part. Bones come in different shapes and sizes, and none of them has the geometry and gross morphology of an ideal mechanical test specimen. Due to a lack of systematic examinations it is not clear, which bone of the rat skeleton offers ideal conditions for determining mechanical properties during three-point bending.

This examination focuses a systematic study of the biomechanical and radiological characteristics of usable bones of the male Wistar rat to identify the optimal bone, at least from a biomechanical point of view, and to facilitate an accurate, unbiased sample size calculation accordingly.

## Methods

### Animals

20 male Wistar rats between 10 and 38 weeks of age with a mean body weight of 476 ± 160 g (range between 240 and 720 g) were euthanized according to the recommendation of the European Union using a threefold overdose of Phenobarbital (90 mg/kg, i.v.). Subsequently the upper and lower limbs were dissected and both femora (*n* = 40), tibiae (*n* = 40) and humeri (*n* = 40) were harvested. Surrounding skin, muscle, and other soft tissues were removed. The bones were fixed and stored in 70% methanol at 4 °C.

### High resolution dual energy X-ray absorptiometry (DXA)

Determination of bone mineral content was accomplished using a high-resolution DXA scanner (pDEXA Sabre, Norland/Stratec, Stratec Medizintechnik, Pforzheim) [15].

For all explanted bones (femur, tibia and humerus) bone mineral content (BMC) and lean mass were depicted; the resolution was 100 µm × 100 µm (acquisition time: 5 mm/s, total time 40 min). Bones were kept in cell culture dishes (Petri) and throughout the experiments in aquatious solution. Immediately after DXA scanning, the length of the bones was assessed with a calliper.

### Peripheral quantitative computed tomography (pQCT)

Before each sequence, specimens were put into a vacuum chamber for 20 min to remove excessive air. Thereafter the bones were analysed by a high resolution pQCT scanner (pQCT-M Research, Stratec Medizintechnik, Pforzheim) [15, 16]. After generating a quick-view (Scout-View) it was possible to acquire transverse section images with a thickness of 500 µm and a spatial resolution within the image plane of 100 µm at the diaphysis and the distal and proximal metaphysis.

Based on the scout-view, each bone’s individual length was designated 100%. The transection positions for the femur were defined at the distal femur metaphysis at 15, 17.5 and 20% of the femoral bone length, at the middle of the diaphysis at 40, 42.5 and 45% and proximally at 64, 66.5 and 69%. The transection positions for the tibia were defined distally at 10, 12.5 and 15%, at the diaphysis 45, 47.5 and 50% and proximally at 79.5, 82 und 84.5%. The humeri were measured distally at 17, 19.5 and 22%, midshaft (diaphysis) at 52, 54.5 and 57% and proximal at 79.5, 82 and 84.5% of the bone’s length. At all sites, an average was calculated from the three cross section images. Transection positions corresponded to the positioning of bearing- and loading-bars during the subsequent three-point-bending test.

Evaluation of the cross section images and analysis of qualitative variables was carried out with the software provided by the manufacturer. For separate calculation of trabecular, cortical and subcortical bone properties, peelmode 1 was used on one hand with a threshold of 600 mg/mm and peelmode 2 on the other (40% surface with lowest density = trabecular compartment) [15, 16].

The metaphyseal mineral content, surface and density of the trabecular and subcortical compartments were assessed for each bone. At the middle of the diaphysis the cortical mineral content, the cortical bone cross section surface and its percentage of the whole cross section surface were measured, as well as cortical density and thickness of the cortical bone, all of which are supposed to serve as a degree of mechanical strength of the bone during bending.

### Mechanical testing (Fig. 1)

Subsequently the bones were subjected to three-point bending (ap-direction femur and humerus, pa-direction tibia) using a Zwick material testing machine (Zwicki Line Z2.5, Zwick/Roell, Ulm, Germany). Positioning for three-point-bending corresponded to the pQCT assessment, with the force transmission at the evaluated middle position between the distal and proximal site. Bearing- and loading-bars had a rounded tip with a diameter of 2.5 mm. The distance between the bars was adapted relative for each bone as specified before. All bones were loaded until failure with a persistent test velocity of 5 mm/min. Meanwhile a load–displacement diagram was recorded every 0.1 s and thereby failure load was determined. Reaction forces were measured using a load cell for up to ±2.5 kN (Klasse 0.05, A.S.T. GmbH, Dresden, Germany). The stiffness was defined as linear regression of the force/displacement graph using the software testXpert V12 (Zwick/Roell, Ulm, Germany).

**Fig. 1:**
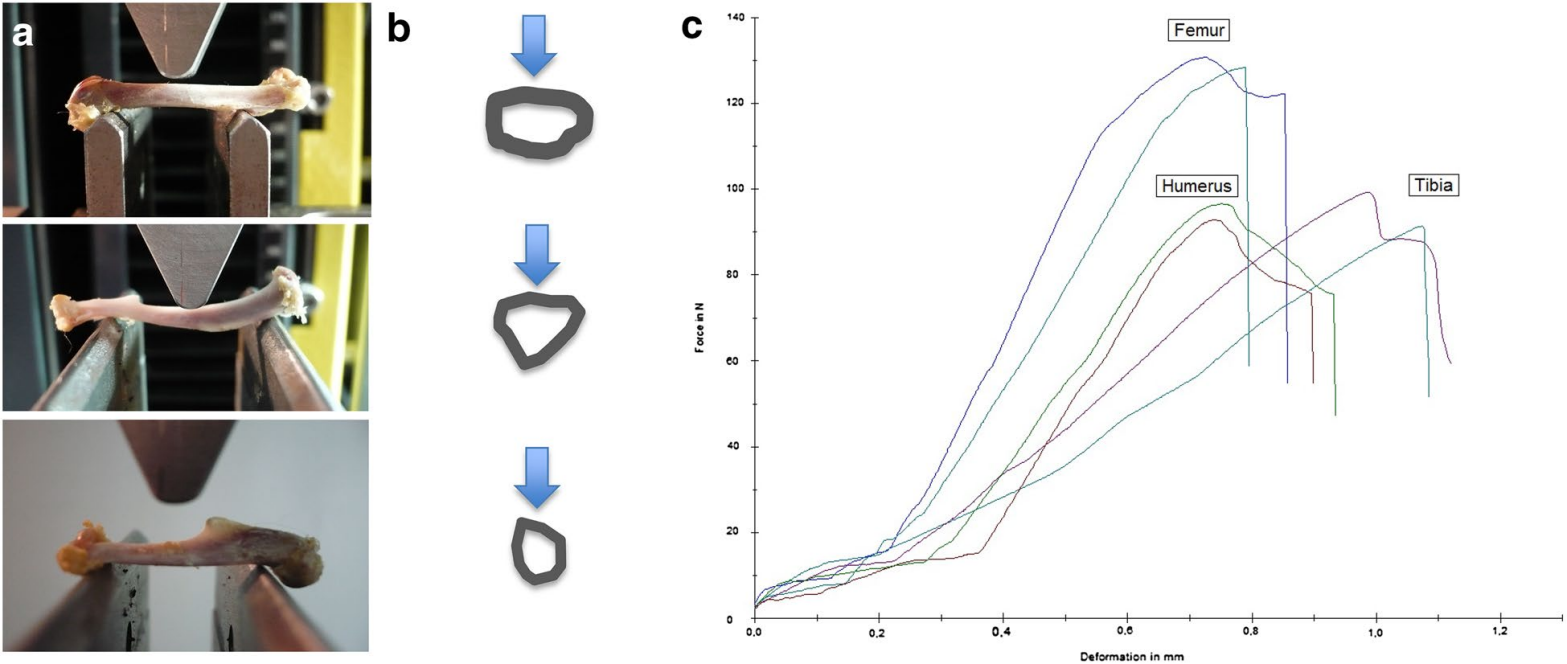
Biomechanical setup, diaphyseal bone cross sections and load–displacement diagram. First column **a**: Setup of 3-point bending for femurs, tibiae and humeri. Individual adjustment of breaking and loading bars for each bone specimen correspond to pQCT-measurement areas. Femurs and humeri are loaded in ap-direction, tibiae in pa-direction. Second column **b**: Schemes of cross-sectional pQCT-images at the level of the loading intender (femur, tibia and humerus). Arrows mark the direction of the applied force. Load–displacement diagram of the six tested bones of one individuum (**c**). *X* axis shows the deformation in mm, *y* axis the reaction forces in *N*. The fracture-curves of both sides were very similar and characteristically for the bone-subtype tested

### Statistical analysis

The acquired data was collected and analysed using GraphPad Prism 5 (GraphPad Software, La Jolla, CA, USA). As quantitative data showed no severe deviations from the normal distribution, descriptive statistics are given by mean ± standard deviation. Percentage variation (CV%) was calculated as CV% = SD/mean × 100.

Group comparisons were performed either by One-way ANOVA followed by Tukey’s test or by Student’s *t* test. *p* values < 0.05 (significance level *α* = 0.05) were declared as statistically significant different. The correlations were determined by the slope of the linear regression graph (including the coefficient of correlation *R*^2^ and the 95% confidence intervals).

## Results

Absolute values of the radiological parameters assessed are summarized in Table 1. We saw neither significant differences between left and right sides, nor a specific handedness concerning radiologic parameters. Mean side differences of the BMC were relatively small (only 1–3% measured by DXA and 2.5–5% by pQCT).

**Table 1:** Summary, radiological data assessed by DEXA and pQCT: means and standard deviations of BMC (g/cm^3^), TOT CNT (total content) and CRT CNT (cortical content)

	BMC (g/cm^3^)	TOT CNT	CRT CNT	Δ right/left BMC	Δ right/left TOT CNT	Δ right/left TOT CNT	*p* value
Femur	0.56 ± 0.20	11.76 ± 3.45	10.78 ± 3.21	0.012 ± 0.033	0.599 ± 1.050	0.276 ± 0.375	n.s.
Tibia	0.41 ± 0.15	8.57 ± 2.57	7.90 ± 2.41	0.007 ± 0.007	0.213 ± 0.177	0.194 ± 0.160	n.s.
Humerus	0.26 ± 0.09	7.65 ± 2.37	7.07 ± 2.21	0.003 ± 0.002	0.276 ± 0.375	0.242 ± 0.356	n.s.

No significant differences between the right and the left sides

**Fig. 2:**
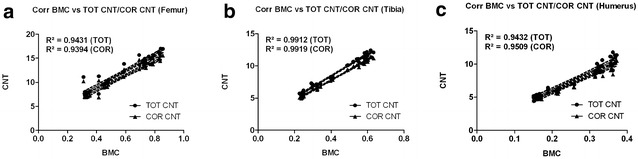
Correlation graphs (Bivariate Scattergrams with regression lines and 95% confidence bands). **a** Correlation graphs of BMC (DEXA, g/cm^3^) vs. TOT CNT and CRT CNT (pQCT) for femurs. **b** Correlation graphs of BMC (DEXA, g/cm^3^) vs. TOT CNT and CRT CNT (pQCT) for tibiae. **c** Correlation graphs of BMC (DEXA, g/cm^3^) vs. TOT CNT and CRT CNT (pQCT) for humeri. In summary high correlations between both radiological methods could be achieved. *BMC* bone mineral content, *TOT CNT* total content, *CRT CNT* cortical content

Overall, bone mineral content (BMC) assessed by DXA and pQCT [total content (TOT CNT), cortical content (CORT CNT)] showed high correlations between each other (BMC vs. TOT and CORT CNT: *R*^2^ = 0.94–0.99) (Fig. 2).

**Fig. 3:**
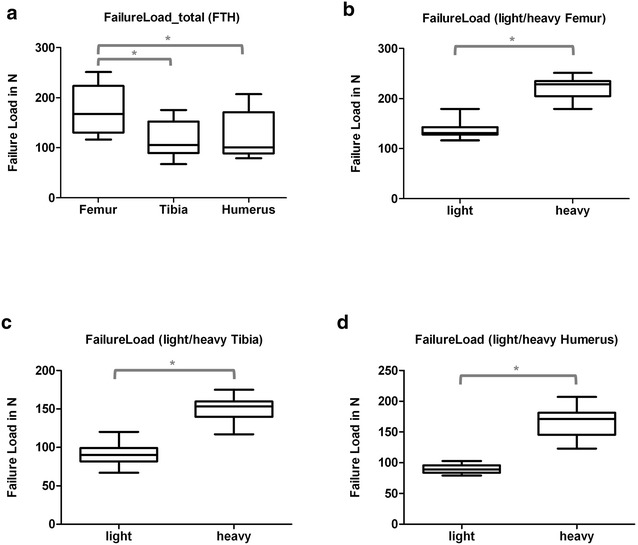
Box plots, failure loads (*N*) for al tested bones (**a**) and for each bone-type separated into specimens of light (< 400 g) and heavy (< 400 g) animals (**b**–**d**). * Indicates significant difference. **a** Summary (Group comparisons by One-way ANOVA, Tukey’s test). **b** Femur (Group comparisons by *t* test). **c** Tibia (Group comparisons by *t* test). **d** Humerus (Group comparisons by *t* test)

**Fig. 4:**
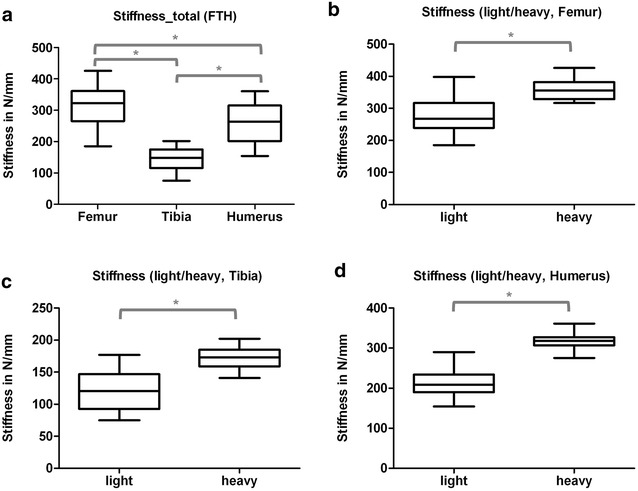
Box plots, stiffness (N/mm) for al tested bones (**a**) and for each bone-type separated into specimens of light (< 400 g) and heavy (< 400 g) animals (**b**–**d**). * Indicates significant difference. **a** Summary (Group comparisons by One-way ANOVA, Tukey’s test). **b** Femur (Group comparisons by *t* test). **c** Tibia (Group comparisons by *t* test). **d** Humerus (Group comparisons by *t* test)

The load–displacement diagram showed a typical curve for each type of bone (Fig. 1). Femurs and humeri conducted quite similar concerning stiffness, the slope of the tibial curves was generally smaller. Interindividual variance in failure loads and stiffness of the bones was notably and counted up to as much as one-third (Table 2). Due to distinct biomechanical characteristics, we divided our population in two subgroups: Light (skeletally immature) rats under 400 g (*N* = 11, 22 specimens of each bone, age < 16 weeks) and heavy (mature) rats over 400 g (*N* = 9, 18 specimens of each bone, age > 16 weeks). The division caused more consistent results in both groups. The smallest interindividual differences were observed in failure loads of the femura (CV% 8.6) and tibiae (CV% 10.7) of heavy animals, whereas light animals showed good consistency in the failure loads of the humeri (CV% 7.7). Most consistent results of both sides (left vs. right) in failure loads were provided by the femora of light animals (mean difference of 4.0 ± 2.8%); concerning the stiffness, humeri of heavy animals showed the largest consistency (mean difference of 6.2 ± 5.0%) (Table 2, Figs. 3 and 4). The single values of each radiological and biomechanical measurement are summarized in the additional data sheet (Additional file 1).

**Table 2:** Summary, biomechanical parameters: means and standard deviations of failure loads (*N*) and stiffness (N/mm) for femurs, tibiae and humeri, total and divided into subgroups of light (< 400 g) and heavy (> 400 g) animals

	Failure load (*N*)	Failure load (*N*) Light/heavy	*p* value failure load Light/heavy	Failure load (*N*) Δ right/left	Stiffness (N/mm)	Stiffness (N/mm) Light/heavy	*p* value stiffness Light/heavy	Stiffness (N/mm) Δ right/left
Femur (*n* = 40)								
Light (*N* = 22)	175.4 ± 45.23	138.1 ± 16.38	< 0.0001	5.6 ± 3.91	315.6 ± 63.00	280.8 ± 59.85	< 0.0001	37.4 ± 24.68
Heavy (*N* = 18)		221.0 ± 18.95		16.0 ± 6.91		358.1 ± 34.64		43.1 ± 16.22
Tibia (*N* = 40)								
Light (*N* = 22)	117.1 ± 33.94	89.6 ± 13.25	< 0.0001	9.8 ± 9.48	143.8 ± 36.99	120.7 ± 32.16	< 0.0001	18.7 ± 12.76
Heavy (*N* = 18)		150.6 ± 16.14		9.4 ± 10.95		171.9 ± 18.28		17.2 ± 14.72
Humerus (*N* = 40)								
Light (*N* = 22)	124.6 ± 41.13	90.23 ± 6.97	< 0.0001	5.5 ± 5.09	260.5 ± 59.97	213.6 ± 35.06	< 0.0001	14.4 ± 7.04
Heavy (*N* = 18)		166.5 ± 20.79		13.2 ± 7.58		317.7 ± 20.42		19.7 ± 15.92

**Fig. 5:**
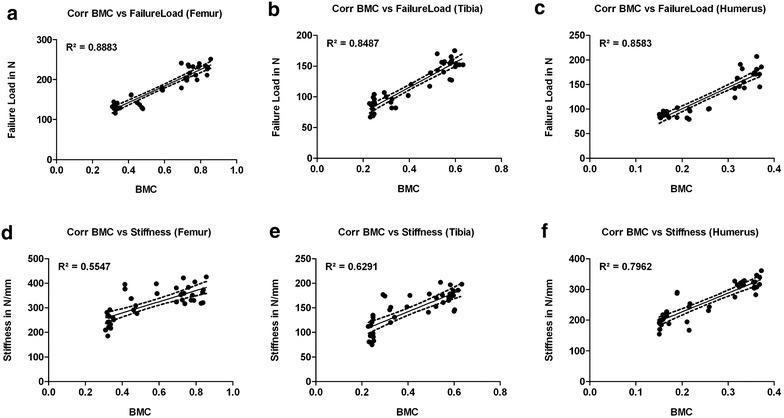
Correlation graphs (Bivariate Scattergrams with regression lines and 95% confidence bands). First line: correlation of the BMC with failure loads for femurs (**a**), tibiae (**b**) and humeri (**c**). In general, strong correlations of the BMC with failure loads could be observed. Second line: correlation of the BMC with stiffness for femurs (**d**), tibiae (**e**) and humeri (**f**). Here, only moderate correlations could be shown, except for the humerus (**f**)

In general, the failure loads showed strong correlations to the BMC (*R*^2^ = 0.85–0.88) whereas stiffness correlated only moderate, except for the humerus (BMC vs. stiffness: *R*^2^ = 0.79) (Fig. 5).

### Humerus

**Fig. 6:**
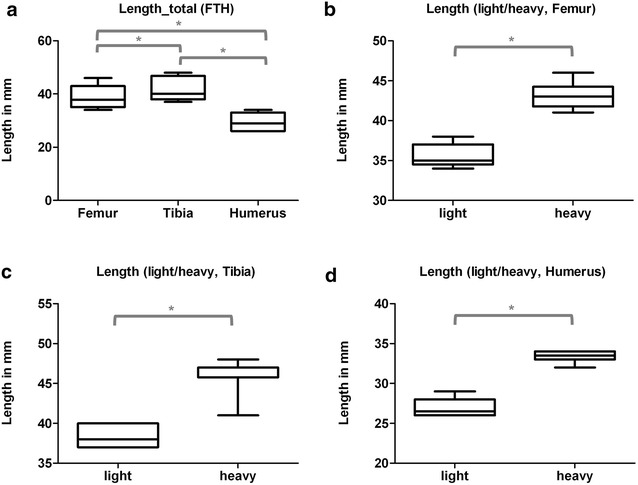
Box plots, length (mm) for al tested bones (**a**) and for each bone-type separated into specimens of light (< 400 g) and heavy (< 400 g) animals (**b**–**d**). * Indicates significant difference. **a** Summary (Group comparisons by One-way ANOVA, Tukey’s test). **b** Femur (Group comparisons by *t* test). **c** Tibia (Group comparisons by *t* test). **d** Humerus (Group comparisons by *t* test)

As proposed, the humerus revealed the shortest bone of the tested specimens. Length varied from 26 to 34 mm (mean 29.88 ± 3.33 mm) with only marginal differences between the left and the right side (1 mm in 3 cases) (Fig. 6). Especially heavy animals showed a very small length range in humeri. Notably, the percentual deviation by the DXA for the BMD (left vs. right) was lowest, (1.1 ± 2.9%) suggesting a particularly homogenous picture (Table 1).

**Fig. 7:**
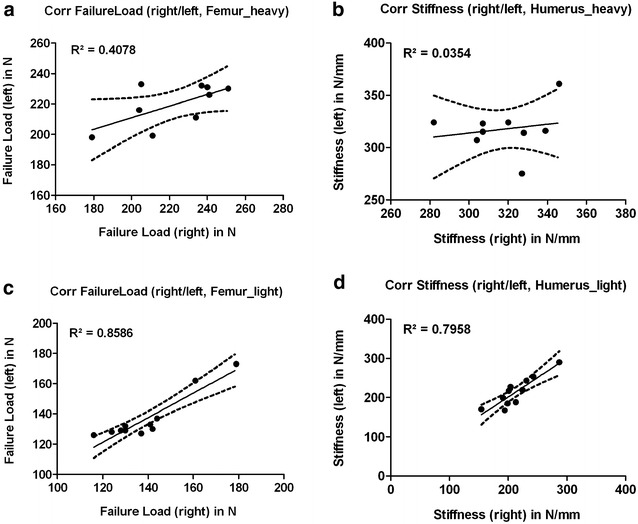
Correlation graphs (Bivariate Scattergrams with regression lines and 95% confidence bands). Left column (**a**, **c**): correlation of the failure loads of the left and the corresponding right side for femurs of heavy animals (**a**) and light animals (**c**). Whereas the correlation in heavy animals was weak and almost random-like, light animals showed a strong correlation. Right column (**b**, **d**): correlation of the stiffness of the left and the corresponding right side for humeri of heavy animals (**b**) and light animals (**d**). No correlation in heavy animals, the humerus of light animals was the only bone reaching an acceptable correlation of the right and the left sides

Concerning biomechanics, failure loads of the bones of light animals and the stiffness of the bones of heavy animals showed peculiar, interindividual constancy, side differences were between 6 and 8% throughout the groups (Table 2, Figs. 3 and 4). In general, the correlation between the left and the right side of one individuum concerning failure load or stiffness was rather weak. In humeri, the bone-stiffness of light animals reached an acceptable value (*R*^2^ = 0.79) (Fig. 7).

However, correlation of the densitometric techniques and the failure loads was high and the humerus was the only bone showing a good correlation of the stiffness and the BMC asides (Fig. 5).

### Tibia

Tibiae were the longest bones, actually ranging from 37 to 48 mm (mean 41.8 ± 4.12 mm). 4 bones differed from their respective counterpart by 1 mm (Fig. 6). Although side differences of more than 0.5 mm were rare, heavy rats showed a relatively large length range in comparison to other bones examined (Fig. 6).

Interindividual variance (CV%) in failure loads of the tibiae was less than in humeri (29%) and could be reduced by subgroup-forming to 14.8% in small and 10.8% in large animals. Interestingly both groups showed comparable variability (Table 2, Figs. 3 and 4).

As in humeri and femurs, we observed a high correlation of the failure load to BMC (Fig. 5).

### Femur

Femurs were observed to provide the largest length-range (34–46 mm totally, mean 38.9 ± 4.12 mm). Unlike humeri and tibiae, many paired bones were different in length (altogether 6 rats, 2 differed by 1 mm, another 2 by 2 mm and 2 rats by even 3 mm). Light rats averaged 35.5 mm, heavy rats 43.1 mm in length. Both subgroups showed comparable consistency (±1.35 mm vs. ± 1.57 mm) (Fig. 6).

Interindividual variance (CV%) in failure load was 25.8% in total and was reduced to under 8.6% if small animals (under 400 g) were excluded. Light animals had a slightly higher interindividual variance with 11.9% (Table 2, Fig. 3). Intraindividual variance (left vs. right) in femurs of light rats was the lowest observed (4 ± 2.8%) (Table 2), heavy rats showed the best correlation of the failure load to the BMC (Fig. 5).

Solely in femurs of light animals the correlation in failure loads between the left and right side was high (*R*^2^ = 0.86), in heavy animals the predictive power between the left and right side was only 40.8% (Fig. 7).

## Discussion

Though biomechanical testing of rat bones plays an important role in preclinical experimental models concerning fracture healing or osteoporosis [5, 17, 18], systematic examinations focusing the biomechanical characteristics of different skeletal locations of the rat are sparse. Our main intention was the assessment of the mechanical properties of the different long bones of the rat skeleton considering both—the accuracy and the consistency of the results achieved using the three point bending test. A comparable research setup has only been performed in a study by Schriefer et al. [13] systematically investigating different bones of the mouse skeleton potentially available for biomechanical testing. Because of the high aspect ratio, the low variability and a minimal measurement error, the authors proposed the radius as an ideal test specimen in three-point bending of the mouse skeleton. This might be true respecting the biomechanical view but may be of concern under practical considerations, at least in interventional models.

From a biomechanical standpoint, a preferable bone for bending tests is one that has a large length to width ratio and a consistent cross-sectional shape along its entire length. To calculate the correct bone tissue properties from a bending test, the bone’s aspect ratio (the ratio of the length to width) should be over twenty [19]. If the bone is short and stout the bending test will generate a larger shear deformation in addition to bending thus reducing the value of Young’s modulus derived from the test. Hence, a bone that best resembles a long, narrow tube, and therefore, has a large aspect ratio generates a better test specimen. Another important factor directly influencing biomechanical measurements is ring-type deformation [13]. When the cortex of the tested bone is thin, it deforms oval under pressure as caused by three-point bending. Accordingly, the measured displacement is not alone due to bending and will draw impact on the actual results. Biomechanically, the ideal bone for three-point bending is a long bone with a constant and thick cortical layer.

Fracture or osteosynthesis models on the other hand, demand either surgical or at least interventional access and a certain size of the bone to perform those interventions in a reasonable setting. This consideration reduces available long bones to the femur, the tibia and the humerus, all of which were subject of our investigation.

Numerous fracture, critical size defect or osteosynthesis studies in rats have been published using the femur or the tibia [17, 18, 20–22]. Only in a few studies, the humerus has been subject to interventional experiments [23, 24]. To the best of our knowledge, no other than the above-mentioned rat bones have ever been used in interventional studies needing a fracture, surgery or stabilisation at the side of the bone.

Especially rodent femurs and tibiae are suitable for experimental studies and have each certain benefits and disadvantages. The femur is surrounded by a thick muscle layer complicating the generation of a standardized fracture. Thus, the good soft tissue coverage allows the use of implants with acceptable complication- and reduced infection rates. Furthermore, by far the most fracture models are established in the femur offering the possibility for comparisons between different studies. The thin soft tissue mantle around the tibia on the other hand allows easy access and especially dedicates this bone for closed fracture models with or without intramedullary stabilisation devices [25]. There is a risk of generating a concomitant fibular fracture which changes a stable into a rather unstable model and often implicates secondary infections in case of plate- or fixateur-application [26]. From a surgical standpoint, most authors prefer the femur in studies where interventions occur directly at the bone [20, 27].

Some specialities of the different bones examined in our study could draw impact on future experimental designs. Largely, no significant difference between left and right bones, nor a specific side preference in sense of handedness in radiologic parameters was evident. Hence, pairing of specimens or evaluating the experimental side relative to the contralateral healthy side seems feasible. The fracture-curves in the load–displacement diagram were specific for each bone. Femurs and humeri showed almost the same stiffness (round cross-section). Tibiae (triangular cross section) showed lower stiffness values, as evident by the more levelled curves. The humerus of the rat is short and stout. Despite the reduced aspect ratio, the failure loads of light animals’ bones and the stiffness of heavy animals’ bones showed remarkable consistency as does the BMD of the left vs. the right side of one individual. Intraindividual variance of failure loads in femurs of light rats was the lowest observed. Heavy rats showed the best correlation of the failure load to the BMC.

Interestingly, the stiffness as a second biomechanical parameter correlated only moderately with DXA and pQCT measurements regarding the humeri and even less regarding the tibiae and femurs. This might be due to the fact that both, the DXA and the pQCT measure the mineralized compartment of the bone, which reflects the failure load biomechanically, whilst the stiffness shows at least in part properties of the liquid and the fibrous tissue of bone [14, 28].

Strong, positive correlations between the biomechanics of the bone and the microstructural parameters obtained by the pQCT and the DXA have been reported for femurs of inbred mice [6] and for the human radius [29], both emphasizing the predictive value of the radiologic analysis. Bagi et al. [7] evaluated the cortical microstructural parameters of the unfractured rat femur obtained by µCT, pQCT and DXA and demonstrated, for some of them, their ability to predict cortical bone strength. In comparison to our results, their coefficients of determination (*R*^2^), regardless of the actual method or parameter, remained relatively low (e.g. correlation between bone strength of the femoral midshaft and bone mineral density of the whole femur determined by DXA: *R*^2^ = 0.34; in our study *R*^2^ = 0.89) [7]. They used a fix span of 15 mm in their biomechanical bending setup; this might indicate that an individualized, biomechanical setting, as applied in our study, generates a better correlation between biomechanical and radiological parameters.

Biomechanical testing protocols vary between different studies. Published techniques have included compressive loading (push), tensile testing (pull), torsional testing and bending [5, 12]. As the type of loading should reflect functionally applied forces for the selected bone, most long bones are either tested by torsion or bending [30]. A consideration to choose either one or the other is that intact long bones fail in a brittle manner during torsional tests in comparison to bending [31]. Hence, torsion should be avoided if perturbation alters bone ductility. Bending tests on the other side are straightforward and can be performed in a three-point or a four-point setting. Of those two, three-point bending is a simple and reproducible test, making it the preferred method even for unexperienced researchers [5, 8, 12, 14]. Thus, most of the biomechanical data published to date has been assessed via three-point bending either of the femur (about 2/3) or the tibia (1/3). The use of the humerus remains exceptional. In 2017, 22 small animal studies used three-point bending in their experimental setup. In three-point bending, fractures generally occur under the center loading point, which enables accurate correlation with, e.g. pQCT measures determined at that known area. If applied in fracture healing studies, the callus can be evaluated exactly at the given point—as long as the callus is hard or mature enough to sustain the loading intender. The direction of the applied load has an impact on the biomechanical behaviour of the bone in accordance to the cross-sectional geometry of the specimen. Thus, loading in direction of skeletal adaption to locomotive loading should provide most accurate results [32].

Four-point bending fixtures are difficult to fabricate to ensure that all four loading points contact the bone [12]. In four-point bending, the fracture occurs at any location between the middle points. Like in torsional testing, we thus measure the weakest point of the whole bone rather than the mechanical competence of the callus and produce a higher variance methodologically than in three-point bending [5].

Adjusting biomechanical measurements for body size (weight, BMI, length of the animal) has been proposed for mouse bones and is a feasible though elaborate measure to reduce variation of results [33]. Interestingly, the biomechanical behaviour of different rodent bones we investigated changed somewhat with the animal’s weight that furthermore corresponds well to the age or the skeletal maturity of the animal. Certainly there will be an effort to evaluate a homogenous cohort of animals in the experimental situation concerning fracture healing studies, though knowledge about the specialities of each bone according to skeletal maturity could be of interest and could be integrated in the experimental design. With respect to our investigations, femurs of heavy, skeletal mature animals produced constant and predictable biomechanical results with a low interindividual variance. By contrast, correlation of the failure loads of right to left femurs was better in small rats, which would dedicate those to experimental designs where the experimental side is evaluated relative to the healthy bone. Of note, the humerus of light animals showed the most homogenous patterns biomechanically, with age and maturity the variance grew concordantly.

Transferability of our data is potentially limited by the fixation method used. Although the morphological, biomechanical or mineralogical characteristics of trabecular bones have been proven not to be altered by this treatment [34], we do not know if this statement can be transferred to cortical specimens. Anyway, as all bones were treated equally and the comparisons between the groups are of far more significance than the absolute values that might have been altered, this systematic failure should not compromise the main conclusions drawn by our study. The direction of loading is a crucial factor substantially altering biomechanical characteristics of bone [32]. As we loaded our bones solely in ap resp. pa direction, we do not know whether our conclusions are applicable in other loading scenarios. All experiments were conducted in bones of male Wistar rats. Naturally, we do not know if other rat-strains would offer comparable biomechanical or radiological conditions.

Total variation of biomechanical measurements in animal fracture studies is composed by methodological and biological variation. Reducing both thus increases the statistical power of the study.

The femur of mature specimens showed the most accurate and consistent results in failure loads of the male Wistar rat, together with the common experimental use enabling comparisons among different studies, identifying this bone as the biomechanically ideal test specimen in three point bending in ap direction. This can be explained by the combination of a superior aspect ratio and a round and long, straight morphology, which satisfies the beam criteria more than other bones tested.

## Additional file

**Additional file 1: Table S1.** Data-summary: Radiological and biomechanical results of each bone.
