# Supplementary material for: Whole bone testing in small animals: systematic characterization of the mechanical properties of different rodent bones available for rat fracture models
Source: Eur J Med Res. 2018 Feb 14;23:8. doi: 10.1186/s40001-018-0307-z (PMC5813325; doi:10.1186/s40001-018-0307-z)
Supplement: Supplementary file 1 — Additional file 1: Table S1. Data-summary: Radiological and biomechanical results of each bone. [file 40001_2018_307_MOESM1_ESM.pptx]

## Slide 1
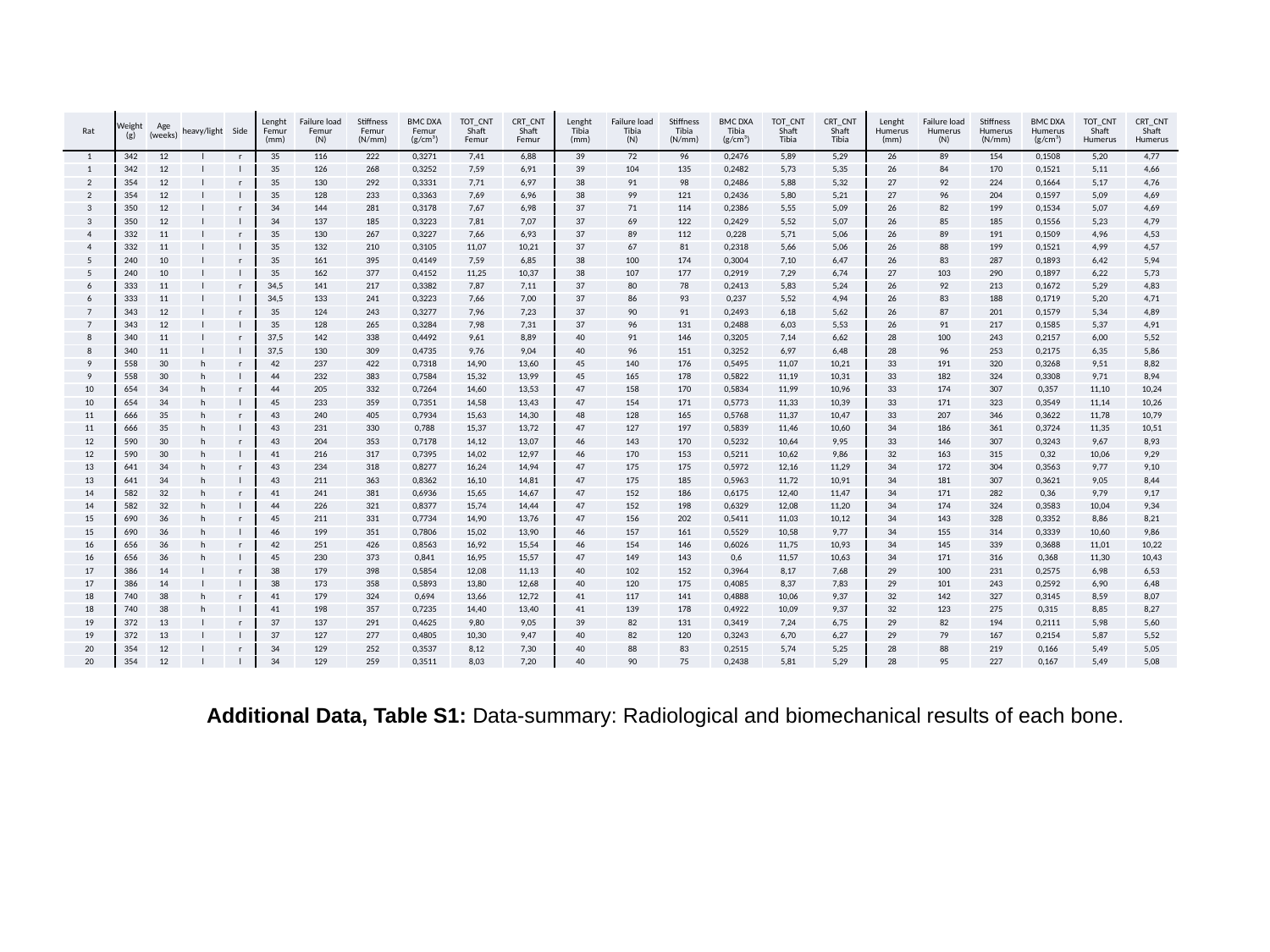

| Rat | Weight (g) | Age (weeks) | heavy/light | Side | Lenght Femur (mm) | Failure load Femur (N) | Stiffness Femur (N/mm) | BMC DXA Femur (g/cm³) | TOT\_CNT Shaft Femur | CRT\_CNT Shaft Femur | Lenght Tibia (mm) | Failure load Tibia (N) | Stiffness Tibia (N/mm) | BMC DXA Tibia (g/cm³) | TOT\_CNT Shaft Tibia | CRT\_CNT Shaft Tibia | Lenght Humerus (mm) | Failure load Humerus (N) | Stiffness Humerus (N/mm) | BMC DXA Humerus (g/cm³) | TOT\_CNT Shaft Humerus | CRT\_CNT Shaft Humerus |
| --- | --- | --- | --- | --- | --- | --- | --- | --- | --- | --- | --- | --- | --- | --- | --- | --- | --- | --- | --- | --- | --- | --- |
| 1 | 342 | 12 | l | r | 35 | 116 | 222 | 0,3271 | 7,41 | 6,88 | 39 | 72 | 96 | 0,2476 | 5,89 | 5,29 | 26 | 89 | 154 | 0,1508 | 5,20 | 4,77 |
| 1 | 342 | 12 | l | l | 35 | 126 | 268 | 0,3252 | 7,59 | 6,91 | 39 | 104 | 135 | 0,2482 | 5,73 | 5,35 | 26 | 84 | 170 | 0,1521 | 5,11 | 4,66 |
| 2 | 354 | 12 | l | r | 35 | 130 | 292 | 0,3331 | 7,71 | 6,97 | 38 | 91 | 98 | 0,2486 | 5,88 | 5,32 | 27 | 92 | 224 | 0,1664 | 5,17 | 4,76 |
| 2 | 354 | 12 | l | l | 35 | 128 | 233 | 0,3363 | 7,69 | 6,96 | 38 | 99 | 121 | 0,2436 | 5,80 | 5,21 | 27 | 96 | 204 | 0,1597 | 5,09 | 4,69 |
| 3 | 350 | 12 | l | r | 34 | 144 | 281 | 0,3178 | 7,67 | 6,98 | 37 | 71 | 114 | 0,2386 | 5,55 | 5,09 | 26 | 82 | 199 | 0,1534 | 5,07 | 4,69 |
| 3 | 350 | 12 | l | l | 34 | 137 | 185 | 0,3223 | 7,81 | 7,07 | 37 | 69 | 122 | 0,2429 | 5,52 | 5,07 | 26 | 85 | 185 | 0,1556 | 5,23 | 4,79 |
| 4 | 332 | 11 | l | r | 35 | 130 | 267 | 0,3227 | 7,66 | 6,93 | 37 | 89 | 112 | 0,228 | 5,71 | 5,06 | 26 | 89 | 191 | 0,1509 | 4,96 | 4,53 |
| 4 | 332 | 11 | l | l | 35 | 132 | 210 | 0,3105 | 11,07 | 10,21 | 37 | 67 | 81 | 0,2318 | 5,66 | 5,06 | 26 | 88 | 199 | 0,1521 | 4,99 | 4,57 |
| 5 | 240 | 10 | l | r | 35 | 161 | 395 | 0,4149 | 7,59 | 6,85 | 38 | 100 | 174 | 0,3004 | 7,10 | 6,47 | 26 | 83 | 287 | 0,1893 | 6,42 | 5,94 |
| 5 | 240 | 10 | l | l | 35 | 162 | 377 | 0,4152 | 11,25 | 10,37 | 38 | 107 | 177 | 0,2919 | 7,29 | 6,74 | 27 | 103 | 290 | 0,1897 | 6,22 | 5,73 |
| 6 | 333 | 11 | l | r | 34,5 | 141 | 217 | 0,3382 | 7,87 | 7,11 | 37 | 80 | 78 | 0,2413 | 5,83 | 5,24 | 26 | 92 | 213 | 0,1672 | 5,29 | 4,83 |
| 6 | 333 | 11 | l | l | 34,5 | 133 | 241 | 0,3223 | 7,66 | 7,00 | 37 | 86 | 93 | 0,237 | 5,52 | 4,94 | 26 | 83 | 188 | 0,1719 | 5,20 | 4,71 |
| 7 | 343 | 12 | l | r | 35 | 124 | 243 | 0,3277 | 7,96 | 7,23 | 37 | 90 | 91 | 0,2493 | 6,18 | 5,62 | 26 | 87 | 201 | 0,1579 | 5,34 | 4,89 |
| 7 | 343 | 12 | l | l | 35 | 128 | 265 | 0,3284 | 7,98 | 7,31 | 37 | 96 | 131 | 0,2488 | 6,03 | 5,53 | 26 | 91 | 217 | 0,1585 | 5,37 | 4,91 |
| 8 | 340 | 11 | l | r | 37,5 | 142 | 338 | 0,4492 | 9,61 | 8,89 | 40 | 91 | 146 | 0,3205 | 7,14 | 6,62 | 28 | 100 | 243 | 0,2157 | 6,00 | 5,52 |
| 8 | 340 | 11 | l | l | 37,5 | 130 | 309 | 0,4735 | 9,76 | 9,04 | 40 | 96 | 151 | 0,3252 | 6,97 | 6,48 | 28 | 96 | 253 | 0,2175 | 6,35 | 5,86 |
| 9 | 558 | 30 | h | r | 42 | 237 | 422 | 0,7318 | 14,90 | 13,60 | 45 | 140 | 176 | 0,5495 | 11,07 | 10,21 | 33 | 191 | 320 | 0,3268 | 9,51 | 8,82 |
| 9 | 558 | 30 | h | l | 44 | 232 | 383 | 0,7584 | 15,32 | 13,99 | 45 | 165 | 178 | 0,5822 | 11,19 | 10,31 | 33 | 182 | 324 | 0,3308 | 9,71 | 8,94 |
| 10 | 654 | 34 | h | r | 44 | 205 | 332 | 0,7264 | 14,60 | 13,53 | 47 | 158 | 170 | 0,5834 | 11,99 | 10,96 | 33 | 174 | 307 | 0,357 | 11,10 | 10,24 |
| 10 | 654 | 34 | h | l | 45 | 233 | 359 | 0,7351 | 14,58 | 13,43 | 47 | 154 | 171 | 0,5773 | 11,33 | 10,39 | 33 | 171 | 323 | 0,3549 | 11,14 | 10,26 |
| 11 | 666 | 35 | h | r | 43 | 240 | 405 | 0,7934 | 15,63 | 14,30 | 48 | 128 | 165 | 0,5768 | 11,37 | 10,47 | 33 | 207 | 346 | 0,3622 | 11,78 | 10,79 |
| 11 | 666 | 35 | h | l | 43 | 231 | 330 | 0,788 | 15,37 | 13,72 | 47 | 127 | 197 | 0,5839 | 11,46 | 10,60 | 34 | 186 | 361 | 0,3724 | 11,35 | 10,51 |
| 12 | 590 | 30 | h | r | 43 | 204 | 353 | 0,7178 | 14,12 | 13,07 | 46 | 143 | 170 | 0,5232 | 10,64 | 9,95 | 33 | 146 | 307 | 0,3243 | 9,67 | 8,93 |
| 12 | 590 | 30 | h | l | 41 | 216 | 317 | 0,7395 | 14,02 | 12,97 | 46 | 170 | 153 | 0,5211 | 10,62 | 9,86 | 32 | 163 | 315 | 0,32 | 10,06 | 9,29 |
| 13 | 641 | 34 | h | r | 43 | 234 | 318 | 0,8277 | 16,24 | 14,94 | 47 | 175 | 175 | 0,5972 | 12,16 | 11,29 | 34 | 172 | 304 | 0,3563 | 9,77 | 9,10 |
| 13 | 641 | 34 | h | l | 43 | 211 | 363 | 0,8362 | 16,10 | 14,81 | 47 | 175 | 185 | 0,5963 | 11,72 | 10,91 | 34 | 181 | 307 | 0,3621 | 9,05 | 8,44 |
| 14 | 582 | 32 | h | r | 41 | 241 | 381 | 0,6936 | 15,65 | 14,67 | 47 | 152 | 186 | 0,6175 | 12,40 | 11,47 | 34 | 171 | 282 | 0,36 | 9,79 | 9,17 |
| 14 | 582 | 32 | h | l | 44 | 226 | 321 | 0,8377 | 15,74 | 14,44 | 47 | 152 | 198 | 0,6329 | 12,08 | 11,20 | 34 | 174 | 324 | 0,3583 | 10,04 | 9,34 |
| 15 | 690 | 36 | h | r | 45 | 211 | 331 | 0,7734 | 14,90 | 13,76 | 47 | 156 | 202 | 0,5411 | 11,03 | 10,12 | 34 | 143 | 328 | 0,3352 | 8,86 | 8,21 |
| 15 | 690 | 36 | h | l | 46 | 199 | 351 | 0,7806 | 15,02 | 13,90 | 46 | 157 | 161 | 0,5529 | 10,58 | 9,77 | 34 | 155 | 314 | 0,3339 | 10,60 | 9,86 |
| 16 | 656 | 36 | h | r | 42 | 251 | 426 | 0,8563 | 16,92 | 15,54 | 46 | 154 | 146 | 0,6026 | 11,75 | 10,93 | 34 | 145 | 339 | 0,3688 | 11,01 | 10,22 |
| 16 | 656 | 36 | h | l | 45 | 230 | 373 | 0,841 | 16,95 | 15,57 | 47 | 149 | 143 | 0,6 | 11,57 | 10,63 | 34 | 171 | 316 | 0,368 | 11,30 | 10,43 |
| 17 | 386 | 14 | l | r | 38 | 179 | 398 | 0,5854 | 12,08 | 11,13 | 40 | 102 | 152 | 0,3964 | 8,17 | 7,68 | 29 | 100 | 231 | 0,2575 | 6,98 | 6,53 |
| 17 | 386 | 14 | l | l | 38 | 173 | 358 | 0,5893 | 13,80 | 12,68 | 40 | 120 | 175 | 0,4085 | 8,37 | 7,83 | 29 | 101 | 243 | 0,2592 | 6,90 | 6,48 |
| 18 | 740 | 38 | h | r | 41 | 179 | 324 | 0,694 | 13,66 | 12,72 | 41 | 117 | 141 | 0,4888 | 10,06 | 9,37 | 32 | 142 | 327 | 0,3145 | 8,59 | 8,07 |
| 18 | 740 | 38 | h | l | 41 | 198 | 357 | 0,7235 | 14,40 | 13,40 | 41 | 139 | 178 | 0,4922 | 10,09 | 9,37 | 32 | 123 | 275 | 0,315 | 8,85 | 8,27 |
| 19 | 372 | 13 | l | r | 37 | 137 | 291 | 0,4625 | 9,80 | 9,05 | 39 | 82 | 131 | 0,3419 | 7,24 | 6,75 | 29 | 82 | 194 | 0,2111 | 5,98 | 5,60 |
| 19 | 372 | 13 | l | l | 37 | 127 | 277 | 0,4805 | 10,30 | 9,47 | 40 | 82 | 120 | 0,3243 | 6,70 | 6,27 | 29 | 79 | 167 | 0,2154 | 5,87 | 5,52 |
| 20 | 354 | 12 | l | r | 34 | 129 | 252 | 0,3537 | 8,12 | 7,30 | 40 | 88 | 83 | 0,2515 | 5,74 | 5,25 | 28 | 88 | 219 | 0,166 | 5,49 | 5,05 |
| 20 | 354 | 12 | l | l | 34 | 129 | 259 | 0,3511 | 8,03 | 7,20 | 40 | 90 | 75 | 0,2438 | 5,81 | 5,29 | 28 | 95 | 227 | 0,167 | 5,49 | 5,08 |
Additional Data, Table S1: Data-summary: Radiological and biomechanical results of each bone.
